# The Perception and Endogenous Modulation of Pain

**DOI:** 10.6064/2012/561761

**Published:** 2012-12-25

**Authors:** Michael H. Ossipov

**Affiliations:** Department of Pharmacology, College of Medicine, University of Arizona, Tucson, AZ 85724, USA

## Abstract

Pain is often perceived an unpleasant experience that includes sensory and emotional/motivational responses. Accordingly, pain serves as a powerful teaching signal enabling an organism to avoid injury, and is critical to survival. However, maladaptive pain, such as neuropathic or idiopathic pain, serves no survival function. Genomic studies of individuals with congenital insensitivity to pain or paroxysmal pain syndromes considerable increased our understanding of the function of peripheral nociceptors, and especially of the roles of voltage-gated sodium channels and of nerve growth factor (NGF)/TrkA receptors in nociceptive transduction and transmission. Brain imaging studies revealed a “pain matrix,” consisting of cortical and subcortical regions that respond to noxious inputs and can positively or negatively modulate pain through activation of descending pain modulatory systems. Projections from the periaqueductal grey (PAG) and the rostroventromedial medulla (RVM) to the trigeminal and spinal dorsal horns can inhibit or promote further nociceptive inputs. The “pain matrix” can explain such varied phenomena as stress-induced analgesia, placebo effect and the role of expectation on pain perception. Disruptions in these systems may account for the existence idiopathic pan states such as fibromyalgia. Increased understanding of pain modulatory systems will lead to development of more effective therapeutics for chronic pain.

## 1. Introduction

Any complete discussion of pain must include not only its somatosensory parameter that allows us to feel nociception, but must also include its motivational and affective qualities with which we experience pain. Pain is defined as “an unpleasant sensory and emotional experience associated with actual or potential tissue damage or described in terms of such damage,” by the International Society for the Study of Pain (IASP) [1]. The sensation of pain is aversive at threshold and serves as an important teaching signal [2, 3]. Acute pain teaches us to recognize and avoid objects or conditions that have the potential to produce injury. The aversive quality of pain in the case of deep tissue injury, infection, or bone fracture promotes immobilization of the affected limb and promotes healing [2–4]. Accordingly, pain serves a protective function and is necessary for survival. However, chronic pain states, such as neuropathic and dysfunctional pain, are considered to be maladaptive in that there is no protective function associated with the pain [5]. Chronic pain is a significant public health concern worldwide and exerts tremendous socioeconomic costs, exceeding $100 billion USD annually [6]. However, it remains an unmet medical need, as the pain medications currently available for the management of chronic pain are inadequate [7–9]. The unsatisfactory management of chronic pain is reflected in the observations that a 50% or greater reduction in pain is achieved in only 30% to 40% of chronic pain patients [8, 9]. It is clear that improving treatments for chronic pain is an important medical priority. A greater understanding of the mechanisms that transduce and transmit nociception, as well as those that underlie the endogenous pain modulatory systems, hopefully will lead to the development of novel therapeutic agents against chronic pain states.

An important factor to consider in the evaluation and management of pain is that it is a highly variable experience among individuals. Whereas pain is generally initiated by activation of nociceptors, that function to detect noxious stimuli capable of producing tissue damage, there is no evidence that the experiential perception of pain is directly correlated with the level of nociceptor activation. The variability of the pain experience along with observations that pain can change in the presence of other factors including past memories, stress, anxiety, distraction, or attention suggests the presence of endogenous pain modulatory systems that can either enhance or inhibit the pain experience [2]. Recent studies have increased our understanding of these pain modulatory systems, and perhaps engagement of these systems can lead to more efficacious therapeutics against chronic pain.

## 2. Peripheral Nervous System (PNS)

Whereas pain refers to an experience with strong emotional, motivational, and cognitive components [4, 10], the process through which potentially damaging stimuli is detected is nociception. Thermal, chemical, or mechanical stimuli are strong enough to be capable of causing tissue damage (i.e.; noxious or nociceptive stimuli), activate specialized sensory neurons, commonly referred to as nociceptors, and to transmit the noxious signal to the central nervous system (CNS). The pseudounipolar sensory neurons have a cell body in the dorsal root ganglion (DRG) or the trigeminal ganglion and axonal projections that terminate in the periphery and the dorsal horn of the spinal cord. Thus, nociceptive stimuli applied at the periphery result in release of excitatory neurotransmitters in the CNS. The nociceptors are either small-diameter thinly myelinated A*δ* fibers or unmyelinated C-fibers. The A*δ* nociceptors are subdivided into the Type I nociceptors, that respond preferentially to strong mechanical or chemical stimuli, but can also respond to high (>50°C) temperatures, and the Type II nociceptors, that respond preferentially to noxious thermal stimuli over mechanical stimuli [11, 12]. Whereas most C-fiber nociceptors are polymodal, responding to thermal, mechanical, and chemical stimuli, there are subpopulations of C-fibers that are selectively heat-sensitive or mechanosensitive, as well as “silent” nociceptors that gain sensitivity to mechanical or thermal stimuli in the presence of inflammation [11].

### 2.1. Transduction Mechanisms

Our understanding of how environmental signals are transduced into activation of sensory fibers received a large impetus by the discovery, characterization, and cloning of transient receptor potential (TRP) channels [13]. The TRP channels consist of 6 transmembrane domains and a pore. Opening the pore allows influx of Na^+^ and Ca^++^, resulting in depolarization and generation of an action potential [13]. The TRPV1 channel, initially identified as the vanilloid receptor 1 (VR1), was the first “pain” channel to be discovered and was characterized by its activation by noxious heat (temperatures >43°C), low pH (<6) and by capsaicin, the ingredient in hot peppers responsible for the burning sensation [14]. The TRPV1 channel is found on most heat-sensitive C and A*δ* nociceptors [11, 13]. The mechanisms through which heat could activate TRPV1 channels were unclear until recently. Animal studies showed that exposure of rat or mouse skin biopsies to noxious heat (>43°C), but not to nonnoxious temperatures, resulted in the release of the oxidized metabolites of linoleic acid 9- and 13-hydroxyoctadecadienoic acid (9- and 13-HODE) into the perfusate in a temperature-dependent manner [15]. These substances, and their metabolites 9- and 13-oxoODE, directly activated TRPV1 [15]. Inhibiting the formation of these metabolites decreased the heat sensitivity of TRPV1 [15]. Infusion of 9-HODE activated cultured trigeminal neurons is obtained from normal mice, but not from mice genetically modified to not express TRPV1 [15]. These studies suggest that the oxidized linoleic acid metabolites are endogenous ligands that activate this channel. Moreover, since the oxidized linoleic acid metabolites are released from injured cells, these substances may also play a significant role in inflammation and hyperalgesia [15, 16]. In addition to the linoleic acid metabolites, the endocannabinoid anandamide, leukotrienes, and 12- and 5-hydroperoxy-eicosatetraenoic acids also directly or indirectly activate TRPV1 [17]. The regulation of the formation of these substances may provide novel targets for the management of chronic pain states. There have been several attempts by the pharmaceutical industry to develop antagonists of TRPV1 as pain therapeutics. Amgen had advanced AMG 517 to Phase I clinical studies, but these studies were terminated when patients developed significant and persistent hyperthermia, with body temperatures reaching 40°C [18]. Abbott developed ABT-102, which produced less hyperthermia than AMG 517, and the hypothermia was ameliorated with repeated dosing [19]. Unfortunately, ABT-102 also impaired the ability to sense warmth and noxious heat and was terminated [19]. Antagonists of TRPV1 advanced by Lilly, GlaxoSmithKline, and AstraZeneca have also failed to successfully complete clinical trials [20]. Recent studies that characterized TRPV1 antagonists based on the ability to block TRPV1 activation by either capsaicin, protons, or heat suggest that it may still be possible to develop clinically useful antagonists that do not produce hyperthermia or block sensation of noxious heat [20, 21].

Other heat-sensitive TRPV channels found on sensory neurons include TRPV2, which is activated at temperatures above 52°C, TRPV3, and TRPV4, which are activated over a range of temperatures from 27°C into the noxious range [11]. More recently, the TRP melastatin 3 (TRPM3) channel, expressed on small-diameter neurons in the DRG and trigeminal ganglion, was found to be activated by noxious heat and was identified as a thermosensitive nociceptor channel [22].

The TRPM8 channel is activated over a range of low (<26°C) temperatures and functions as a detector of environmental cold [23]. Menthol produces its cool sensation by activation of this channel [23]. Studies with knockout mice lacking the TRPM8 receptor showed that responses of nerve fibers and cultured trigeminal neurons to cool temperatures were markedly diminished, as was the avoidance behavior of the mice to cool surfaces [23]. However, both behavioral and electrophysiologic responses were present to temperatures below 10°C, indicating the possible existence of another sensor to noxious cold [23]. The TRPM8 channel is found on A*δ* and C-fiber neurons and is not expressed with the TRPV1 receptor [24].

The TRPA1 channel has been proposed as the transducer for noxious cold, since TRPA1 knockout mice showed diminished behavioral responses low temperatures (i.e., 0°C) [25]. Upregulation of the TRPA1 channel in DRG neurons is necessary for hyperalgesia to cold after inflammation and nerve injury [26]. However, other studies with TRPA1 knockout mice showed that these mice retained sensitivity to noxious cold, indicating that this channel does not detect noxious cold [23, 27]. More recent studies indicate that the TRPA1 channel mediates mechanical and cold allodynia resulting from paclitaxel-induced neuropathy [28, 29]. These conflicting studies indicate that the role of TRPA1 in noxious cold detection remains unsettled. In addition to a potential role in detecting cold, the TRPA1 channel is activated by several numerous pungent chemicals, including isothiocyanates found in horseradish, cinnamaldehyde found in cinnamon, and allicin found in garlic and produces a burning sensation [30]. It is also activated by acrolein and similar volatile irritants and in response to activation of the bradykinin B2 receptor by bradykinin [27]. Evidence exists that the TRPA1 channel contributes to mechanical nociception and to mechanical hyperalgesia [25, 31]. The TRPA1 channel may mediate the hyperalgesic inflammatory responses to environmental irritants and to endogenous algesic substances such as bradykinin [27].

Evidence for a pronociceptive role for TRPA1 is provided by genetic analysis of patients with familial episodic pain syndrome (FEPS) [32]. These patients may develop debilitating pain in the upper body with stress, fatigue, or fasting, but show normal responses to nociceptive stimuli between episodes [32]. Genetic analyses showed that these patients have a point mutation (N855S) in the S4 transmembrane segment of the TRPA1 channel, which results in a 5-fold increase in the inward current upon activation, resulting in a gain of function of the channel [32]. Moreover, since TRPA1 is expressed in peripheral afferent C-fibers that also express TRPV1, it may mediate the phenomenon of “burning cold” [33]. The coexpression of TRPA1 with TRPV1 suggests interactions between these transducing channels could exist to enhance nociceptive signaling. Recent studies in a mouse model of acute pancreatitis demonstrated upregulation of both TRPV1 and TRPA1 along with increased neuronal excitability of visceral nociceptors [34]. The use of antagonists to TRPV1 and to TRPA1 revealed a synergistic interaction between these TRP channels in producing pancreatic inflammation and pain-related behaviors [34]. Thus, the development of TRPA1 antagonists may be useful in the treatment of enhanced pain in pancreatitis and other inflammatory conditions [34, 35]. There are several TRPA1 antagonists in preclinical development for acute, neuropathic, and inflammatory pain [35]. These include trichloro(sulfanyl)ethyl benzamides (Amgen Laboratories), thioaminal compounds (Abbot Laboratories), and purine-based compounds such as HC-030031 and its derivatives (Hydra Laboratories) [35]. None of these TRPA1 antagonists have yet progressed to clinical studies at the present time.

In contrast to the channels sensitive to temperature, the identification of transducers for mechanical stimuli is still unsettled. One reason may be due to difficulties with stimulation protocols used in behavioral and electrophysiologic assays and in extrapolating cellular and tissue assays to nociception [11]. Mechanosensitive channels that belong to the degenerin/epithelial Na^+^ channel family (DEG/ENaC) were identified in *D. melanogaster* and *C. elegans*, and mammalian orthologs of the MEC-2 protein, which modulates activity of invertebrate mechanosensors, have been found to modulate activity of the acid-sensing channels (ASIC) [36]. Although ASIC channels are present on low-threshold and high-threshold mechanoreceptors, genetic deletion of ASIC1, ASIC2, or ASIC3 produced very little change in mechanical sensitivity, and there is no strong evidence that these channels are relevant to mechanical pain [11]. The TRPV2, TRPV4, and TRPA1 channels have been postulated as potential mechanotransducers based on similarities to nonmammalian transducers, but studies with conflicting results indicate the roles of these channels in mechanical transduction remains unclear [11, 36]. Recent studies found that the mouse Piezo1 and Piezo2 proteins are evolutionarily conserved ion channels that may provide mechanotransduction in Drosophila and mammals [37]. The over expression of mouse Piezo1 (MmPiezo1) and MmPiezo2 in mouse, rat, and human cell lines produces 2 distinct mechanically activated ion currents, indicating that these ion channels may be mechanically sensitive [37, 38]. The Piezo2 protein is expressed in mechanosensitive DRG neurons [37]. Moreover, a role in noxious mechanosensation is suggested by a 24% overlap in expression of TRPV1 [37]. Recent studies showed that activation of the bradykinin B2 receptor increased current amplitude and slowed the inactivation of the Piezo2 channel through mechanisms linked to protein kinase A (PKA) and protein kinase C (PKC) [39]. These effects were abolished by PKA and PKC inhibitors, suggesting that Piezo mediates bradykinin-induced, PKC-mediated mechanical hyperalgesia [39].

### 2.2. Sensitization, Adaptation

 Nociceptors demonstrate remarkable plasticity that can amplify tissue signaling. Tissue injury or inflammation causes the release of sensitizing and algogenic agents, such as bradykinin, histamine, prostaglandins, interleukin-1*β* (IL1*β*), tumor necrosis factor (TNF), and NGF [5]. These substances reduce the activation threshold of nociceptors and increase neuronal excitability, resulting in enhanced neuronal firing. Increased activity of nociceptors is manifest as hyperalgesia (exaggerated pain in response to normally painful stimuli) and allodynia (normally nonnoxious stimuli elicits sensations of pain) [5, 40].

In addition to its role in the development and differentiation of sensory neurons, NGF is an important sensitizing agent for peripheral nociceptors. Tissue injury causes an upregulation and increased release of NGF [41, 42]. Peripheral nerve injury results in NGF release from Schwann cells and fibroblasts in the area of the injury [43]. Binding of NGF to the TrkA receptor on primary afferent nociceptors results in phosphorylation of the TRPV1 channel and a rapid sensitization of nociceptors to heat [44, 45]. The signaling cascades that are activated by NGF and lead to phosphorylation of TRPV1 include PKA, PKC, MEK, the MAP kinases, PI3K, and CaMK II [44, 45]. In addition, NGF binding to TrkA produces a rapid facilitation of TTX-resistant sodium currents and suppresses outward potassium currents, resulting in increases nociceptor activity [45]. In addition to its direct effects, NGF can promote peripheral sensitization by eliciting the formation of leukotriene through the 5-lipoxygenase pathway, stimulating chemotaxis of mast cell and their subsequent degranulation, which results in the release of potent sensitizing and algogenic agents (e.g., bradykinin, histamine, serotonin, and NGF), thus resulting in hyperalgesia due to a sustained state of peripheral sensitization [44]. Peripheral sensitization mediated by NGF is also mediated through posttranscriptional mechanisms in addition to its immediate effects. Transport of the NGF/TrkA complex to the cell bodies in the DRG activates signaling cascades that result in upregulation of TRPV1 and of sodium channels [44, 46] and increases the levels of mRNA and of protein for the excitatory neuropeptide transmitters substance P and CGRP [44].

The importance of NGF in pain sensation is also highlighted by rare instances of hereditary sensory and autonomic neuropathies (HSANs) with insensitivity to pain. The HSAN IV and HSAN V conditions result from one of at least 37 mutations of the NTRK1 gene that result in loss of function of the TrkA receptor [47, 48]. These individuals show an absence of skin innervation by A*δ* and C-fibers [47, 48]. HSAN IV is characterized by severe anhidrosis, varying degrees of mental retardation, impaired thermal sensation, and an absence of pain perception [47, 48]. There are 37 different known mutations that produce HSAN IV [47]. Differences in the ability of TrkA to bind to NT3 may dictate the severity of the disorder, as diminished TrkA/NT3 signaling results in axons not reaching the target sites, whereas diminished NGF/TrkA signaling may allow axons to successful reach the target, but degenerate afterwards [48]. A rare mutation of the NGFB gene, coding for NGF, was discovered in a large Swedish family with members expressing HSAN V, characterized by normal sensory perceptions, normal perspiration, and normal cognitive function, but with a loss of pain and temperature sensation [49, 50]. The afflicted individuals suffered pain-free joint destruction and bone fractures and showed a moderate loss of A*δ* fibers and a marked loss of unmyelinated C-fibers [49, 50]. A novel mutation of the gene coding for NGF was found in an Arab family with children that had an inability to perceive pain, but also had anhidrosis and loss of temperature discrimination [51]. This finding indicates that HSAN IV and HSAN V form a spectrum of phenotypic expression of altered TrkA/NGF signaling [51].

Changes in membrane potentials, and consequently neuronal sensitization and generation of action potentials, are mediated through the voltage-gated sodium channels (VGSCs). Of the VGSCs essential for nerve conduction, three, Na_V_1.7, Na_V_1.8, and Na_V_1.9, are predominantly expressed in nociceptors [52]. The tetrodotoxin-(TTX-) sensitive channel Na_V_1.7 generates a fast activating and inactivating current and is crucial for the development of action potentials [52, 53]. Its role in pain transmission was convincingly demonstrated by the identification of several genetic mutations that resulted in increased pain or loss of pain perception [52, 53]. Erythromelalgia, also called erythermalgia, is characterized by episodic burning pain and redness of the extremities and is often precipitated by exercise or warmth [54]. A study of several members that included several generations of 2 Chinese families with hereditary erythromelalgia revealed that the afflicted individuals had one of 2 missense mutations of the *SCN9A* gene, which encodes the *α*-subunit of the Na_V_1.7 sodium channel [55]. Since then, genetic investigations of families where erythromelalgia was present have revealed that this disorder was caused by more than 20 different mutations of the *SCN9A* gene [56–59]. These gain of function mutations lower the threshold to open the channel, and the slow deactivation due to the mutations keeps the channel open for a longer period of time and also increases the current amplitude [54, 59]. Consequently, these alterations in the kinetics of the Na_V_1.7 channel result in hyperactivity of nociceptors [54, 59]. Gain of function mutations of the Na_V_1.7 channel are also the cause of paroxysmal extreme pain disorder (PEPD) which is characterized by unprovoked, paroxysmal pain in the rectal, ocular, or submandibular regions [60]. The mutations causing PEPD occur in, or close to, the inactivation gate and thus slow inactivation, keeping the channel open longer and increasing persistent currents, allowing repetitive firing of nociceptors [60]. The differences in how the mutations affect the channel account for the different presentation of these disorders and the differences in appropriate treatment [60].

Mutations of the *SCN9A* can also result in loss of function of the Na_V_1.7 channel, resulting in congenital insensitivity to pain (CIP) [58, 61, 62]. CIP differs from the HSANs in that there is no other attendant neuropathy [61]. The “index case” linking CIP to a mutation of SCN9A was a 10-year-old Pakistani boy that performed “street theater” by walking on hot coals or stabbing knives through his arms and performing other such stunts who did not appear to feel any pain [63]. In all, 6 individuals from 3 consanguineous families were identified with CIP [63]. Sequence analysis of *SCN9A* revealed that in 2 of the families, 2 different base substitutions were found, and the third had a deletion producing a frame-shift mutation [63]. These mutations caused truncation of Na_V_1.7 and a complete loss of function of this channel [63]. Since that time, as many as 16 different nonsense mutations of *SCN9A* have been identified in families with members that have CIP [53, 58, 61, 64–66]. In addition, an individual with compound heterozygous mutations of *SCN9A* and absence of pain also was recently identified [67]. Recently, 4 different mutations that produce partial, instead of complete, loss of pain sensation have been reported [68, 69]. Because individuals with CIP have absolutely no pain sensation, they suffer from multiple fractures, osteomyelitis, burns, and wounds [53]. They tend to bite their lips and tongue to the extent that surgery is required to repair the damage [53]. Many do not survive childhood because of these injuries [53, 63, 67]. Since the Na_V_1.7 channel is found only on nociceptors, autonomic neurons, and olfactory nerves, these individuals have normal sensory discrimination, can detect sharp versus dull or hot versus cold, and show normal nerve conductance, but have impaired sense of smell [53, 58, 61, 63]. Notably, they also express normal autonomic function, indicating that this channel is not critical for autonomic nerve function [53, 58, 61, 63].

The enhanced pain and the absence of pain produced by the mutations of *SCN9A* demonstrate that the Na_V_1.7 channel clearly has a critical role in the functioning of nociceptors. Accordingly, selective blockade of this channel is a very attractive target for drug development that is being pursued by AstraZeneca, Xenon Pharmaceuticals, and others [70–73]. Xenon Pharmaceuticals reported that, in a pilot study, the Na_V_1.7 blocker XEN402 produced marked and significant pain reduction in patients with erythromelalgia [74].

The Na_V_1.8 channel is TTX-resistant channel and is almost exclusively expressed in nociceptors [75, 76]. It is a rapidly activating and slow inactivating current with rapid repriming and is required for upstroke overshoot of action potentials of nociceptors [77]. The coexpression of Na_V_1.8 with Na_V_1.7 in peripheral nociceptive afferent neurons allows generation of action potentials even when depolarized [77]. Because the Na_V_1.7 channel shows slow closed state inactivation, it responds to small subthreshold depolarizing inputs, raising the membrane potential towards the action potential threshold, thus amplifying these slow depolarizations and, consequently, pain signaling [77–80]. Since the Na_V_1.8 channel remains functional even at depolarized thresholds, it supports repetitive firing of nociceptors [77, 81]. This interaction between these 2 channels is revealed in the different responses of nociceptors and autonomic neurons in patients with erythromelalgia [82, 83]. Studies performed with cultured rat or mouse DRG and superior cervical ganglia (SCG) neurons that were transfected with the L858H or R185H *SCN9A* mutations, which produce erythromelalgia in humans, revealed that the transfected DRG neurons were hyperexcitable, whereas the SCG neurons were hypoexcitable [77, 82, 83]. Transfecting the SCG neurons with the gene for Na_V_1.8 rendered them hyperexcitable [77, 82, 83].

The Na_V_1.9 is also TTX-resistant. It is responsible for maintaining a persistent current close to the resting membrane potential (−70 mV) [84]. It is believed to set the resting potential of nociceptors and can modulate the membrane potential in response to subthreshold stimuli [84]. Nerve injury in animal models of neuropathic pain markedly reduces the expression of Na_V_1.9. However, knock down of this channel does not alter behavioral signs of neuropathic pain in these animal models [75, 85, 86].

### 2.3. Drug Intervention Strategies

Based on promising results in animal models, clinical studies with recombinant human NGF (rhNGF) were initiated with healthy volunteers or patients with diabetic neuropathy [87, 88]. However, rhNGF failed to improve outcome measures in phase III trials, but did cause significant pain and subsequent hyperalgesia at the sites of injection [87, 89]. The iv injection of NGF produced widespread deep aching pain and hyperalgesia with a rapid onset [88]. Similar results were seen in a clinical trial for HIV-induced neuropathy, where rhNGF rapidly produced pain and myalgia after injection [90]. The rapid production of NGF-induced pain strongly supports that the pain and hyperalgesia are due to NGF-induced peripheral sensitization [45].

In part because of this strong evidence indicating a pronociceptive role for NGF, drug development efforts were initiated to develop antibodies to NGF by several pharmaceutical companies. According to the informational database ClinicalTrials.gov, Astra-Zeneca, Johnson and Johnson, Pfizer, and Regeneron jointly with Sanofi-Aventis SA, advanced the NGF antibodies MEDI-578, fulranumab, tanezumab, and REGN475/SAR164877, respectively, to clinical trials. Tanezumab, a recombinant humanized anti-NGF monoclonal antibody, showed efficacy in early clinical trials for lower back pain and for osteoarthritis with a good safety profile [91, 92]. Although tanezumab produced significant improvement against osteoarthritis, the phase III clinical trials were halted when some patients developed serious accelerated degeneration of joints, including hip, shoulder, or knee to the extent that total joint replacement was required in some cases [93, 94]. It was suggested that the progression of the joint deterioration was due primarily to the osteoarthritic condition, and not a direct result of tanezumab, since cynomolgus monkeys receiving multiple doses over a 26-week period showed no signs of any adverse effects of pathology [95]. It is possible that the pain relief resulted in excessive use and subsequent deterioration of the arthritic joint. A clinical hold was placed on fulranumab, currently in phase I clinical testing for osteoarthritis, by the US FDA in late December, 2010, also because of concerns regarding joint degeneration. The clinical hold also halted phase II clinical testing of MEDI-578 and of REGN475/SAR164877 because of the concerns regarding accelerated joint degeneration. On 12MARCH2012, the Arthritis Advisory Committee of the FDA recommended recommencing clinical studies contingent on developing risk minimization strategies for patients that might be at risk of osteonecrosis, as NGF antibodies show considerable therapeutic benefit [96].

## 3. Central Nervous System (CNS)

### 3.1. Ascending Pathways

**Figure 1:**
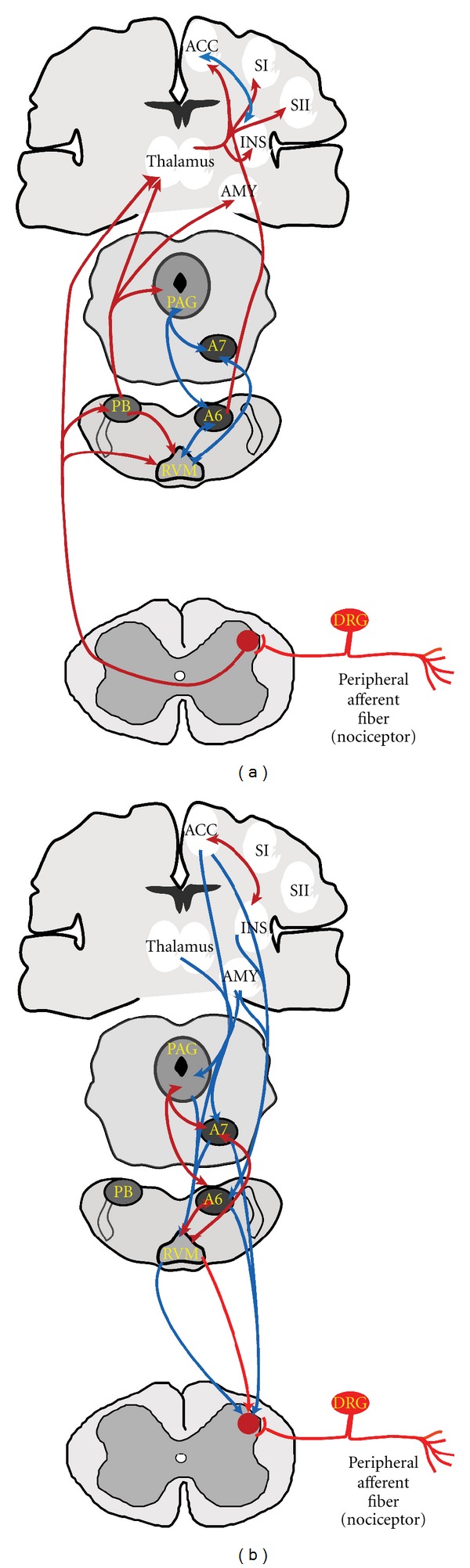
The major ascending (a) and descending (b) pain modulatory systems are illustrated in this schematic representation. Nociceptive inputs enter the CNS at the spinal dorsal horn where primary afferent terminals synapse with second-order projection neurons. The ascending tracts in A are represented in red, and the blue 2-headed arrows indicate bilatateral communications. Descending projections in B are shown in blue, and the 2-headed arrows in dark red indicate bilatateral communications. The light red and blue projections from the RVM to the spinal cord are intended to suggest descending inhibition and facilitation. The regions in the illustration are A6 and A7 noradrenergic nuclei, ACC: anterior cingulate cortex, AMY: amygdala, DRG: dorsal root ganglion, INS: insular cortex, PAG: periaqueductal grey, PB: parabrachial nuclei, RVM: rostroventromedial medulla, SI: primary somatosensory cortex, and SII: secondary somatosensory cortex.

The central terminals of the peripheral sensory fibers enter the CNS through the dorsal horn of the spinal cord or the n. caudalis, as shown in Figure 1(a). The substantia gelatinosa, consisting of laminae I and lamina II outer, receives inputs from myelinated A*δ* nociceptors and the peptidergic unmyelinated C-fiber nociceptors, and most of the nonpeptidergic C-fiber nociceptors terminate on interneurons of the inner lamina II [97]. The deeper laminae, III through V, receive inputs from the large-diameter myelinated A*β* fibers, which normally transmit innocuous sensory inputs. Moreover, the wide dynamic range neurons of lamina V receive inputs from nonnociceptive primary afferents and from A*δ* nociceptors and also receive indirect inputs from C-fibers terminating on lamina II interneurons via multisynaptic projections [98, 99]. Neurons of laminae I and V project along the spinothalamic and spinoreticulothalamic tracts to supraspinal sites such as the thalamus, parabrachial nucleus, and amygdala, where pain signals are processed and sent on to higher cortical centers [98, 99].

The central terminals of the primary afferent neurons release the excitatory neurotransmitters glutamate, substance P and CGRP to activate the second order neurons of the spinal dorsal horn or the n. caudalis [98, 99]. The lamina I neurons that express the putative receptor for substance P, NK1, account for less than 5% of lamina I neurons, but they comprise over 75% of the nociceptive-responsive lamina I neurons that project through the spinothalamic tract (STT) [100, 101]. These neurons are important targets for peptidergic primary afferent nociceptors [100, 101]. In transmission of acute pain, glutamate released from nociceptors acts on the AMPA/kainite glutaminergic receptors of the spinal cord neurons to activate ascending nociceptive signaling [33, 98, 99]. Additionally, noxious stimuli cause release of substance P, eliciting internalization of the NK1 receptor and activating lamina I neurons. Whereas the neuronal targets for CGRP have not been well characterized, spinal administration of CGRP receptor antagonists diminish behavioral signs of pain in rats [102].

Persistent activation of peripheral nociceptors, as seen with inflammation or nerve injury, results in the development of central sensitization, resulting in enhanced nociception (hyperalgesia) and pain elicited by normally nonnoxious stimuli (allodynia) [5, 33, 99]. Central sensitization is characterized by increases in the spontaneous activity, evoked responses and of the receptive field, the presence of neuronal after discharge, and lowered response thresholds of wide dynamic range (WDR) dorsal horn neurons [5, 33, 99, 101, 103, 104]. The augmented release of excitatory neurotransmitters into the spinal dorsal horn under conditions of enhanced peripheral activity results in activation of the NMDA receptor by glutamate, which is not activated under normal conditions [105]. This in turn results in activation of downstream signaling cascades that, by modulating NMDA receptor activity, enhance neuronal excitability [105]. These proexcitatory neuroplastic changes suggest that enhanced pain states are mediated in part by development of long-term potentiation (LTP) [106, 107]. In the sensitized state, normally nonnoxious stimuli (e.g., light brush), elicits NK1 internalization in lamina I neurons, and neurons of deeper laminae, which normally do not show NK1 internalization, develop this response [108, 109]. Increased NK1 internalization is consistent with electrophysiologic and behavioral characteristics of central sensitization. Blockade of the NK1 receptor alone, however, is insufficient to abolish acute or chronic pain [110–112]. However, selective ablation of lamina I neurons that express the NK1 receptor diminished behavioral and electrophysiologic signs of central sensitization in animal models of inflammation or nerve injury without altering responses to acute nociception [103, 113]. Ablating the ascending nociceptive inputs abolished descending pain facilitation, reversing the signs of enhanced pain, and revealing the importance of spinal-supraspinal neuronal circuits in the maintenance of central sensitization and chronic pain states [101, 103, 104, 114]. It is now appreciated that significant descending pain modulatory systems from supraspinal sites exist that can enhance or inhibit pain.

### 3.2. Endogenous Mechanisms

The ability of the brain to modulate pain has long been suspected, but experimental evidence of such pain modulation was acquired only during the past few decades. One of the earliest documented reports suggesting the existence of some kind of endogenous pain inhibition comes from the published experiences of a physician serving in the US Army during the second World War. He noted that a large majority of soldiers with severe wounds reported either no pain or moderate pain and were reluctant to take pain relief medication [115]. What was striking about this observation was that the wounds were nontrivial, consisting of compound fractures of long bones or penetrating wounds of the abdomen, thorax, or cranium [115]. Moreover, these individuals were clearly alert and responsive, and not in shock, leading Beecher to conclude that “strong emotions” block pain [115]. Similar observations of athletes continuing competition despite significant injuries also suggest existence of endogenous pain modulation [116].

An important consideration in the discussion of pain modulation is to recognize that pain is a sensory and affective experience [2]. Whereas the activation of nociceptors and the transduction and transmission of pain-inducing signals to the CNS is considered nociception, the experience of pain includes not only the somatosensory aspect but the motivational and affective components as well [2]. It has been stated that to ignore these components is to look at only part, and not even the most important part, of pain [2, 117]. Advances in imaging techniques greatly enhanced the resolution needed to determine the brain regions that are responsive to pain and regions that might contribute to pain modulation. Cortical processing of pain is conceptualized as consisting of a medial and lateral pain system originating from thalamic nuclei [118]. The medial pain system is generally associated with the affective/motivational aspects of pain and consists of the medial thalamic nuclei, the anterior cingulate cortex (ACC) and the insula [118]. The lateral pain system consists of the primary (SI) and the secondary (SII) somatosensory cortices and the ventroposterior lateral and medial nuclei of the thalamus and is generally thought of as mediating the sensory/discriminative aspect of pain [118]. Recent evidence suggests that the medial pain system also contributes to pain discrimination [119, 120].

This dichotomy in the pain experience is illustrated by several studies. An individual with a poststroke lesion of the right SI and SII cortices reported normal pain sensations to thermonociceptive laser stimulation applied outside the affected area [121]. Application of noxious heat to the affected hand was “clearly unpleasant,” such that the patient wanted to avoid the stimulus, but he would not describe it as “pain,” even when given choices from menus of words describing pain [121]. Thus, whereas the somatosensory discrimination of pain was absent due to the stroke, the unpleasantness associated with the noxious stimuli remained [121]. In a study using PET scans, the application of electrical stimuli that was highly unpleasant to normal control volunteers produced activation of the thalamus and SI in patients in persistent vegetative states [122]. Whereas the control volunteers also showed activation of SII, the insula, and the ACC, these regions were not activated in the vegetative state, as these patients were not thought to have a conscious experience of pain [122]. Animal studies with rats with peripheral inflammation showed that lesions of the hindlimb somatosensory cortex showed diminished behavioral responses to noxious evoked stimuli, but maintained escape/avoidance behavior to the application of noxious stimuli [123].

The converse has been seen with lesions of the ACC. Patients with intractable pain that received cingulotomies reported immediate relief from the “suffering” associated with the pain [124]. They reported that they could still feel the pain, but that it was no longer bothersome [124]. In a study with human volunteers receiving noxious thermal stimuli, PET scans revealed activation of the S1, S2, insula, and ACC [125]. Hypnotic suggestions made to increase or decrease the “unpleasantness” of the stimulus, which remained unchanged in intensity, elicited changes only in the ACC, indicating that the affective component is coded in the ACC but not the SI [125].

Behavioral studies with animals also point to the ACC as important in motivational aspects of pain. Stimulation of the rostral ACC (rACC) with glutamate increased, whereas rACC lesions decreased, the length of time rats would remain in a chamber associated with formalin-induced pai. [3]. This study suggests that pain-induced ACC activation is a powerful aversive “teaching signal” [3]. Lesions of the rACC or blockade of LTP in the rACC in rats with peripheral nerve injury blocked conditioned place preference (CPP) to pain-relieving manipulations but did not abolish responses to evoked nociceptive stimulation [126, 127]. Thus, the motivational aspect to seek pain relief was abolished, whereas the somatosensory component of neuropathic pain was maintained after these manipulations [126, 127]. Other studies showed abnormal excitability of the ACC of rats after nerve injury or formalin-induced inflammation [128].

The role of the rACC in pain-related learning and contextual pain memory was demonstrated in a rodent model of visceral pain. Visceral hypersensitivity induced by anaphylactic rectal inflammation in rats increased the proportion of ACC neurons responsive to colorectal distension and produced upregulation of the NR2B subunit of the NMDA receptor in the rACC [129]. Attenuation of NR2B activation either pharmacologically with antagonists or by introduction of SiRNA to reduce expression of the subunit normalized both the enhanced visceromotor response and the enhanced neuronal responses of the rACC to colorectal distension [129]. Electrical or chemical (glutamate) stimulation of the rACC enhanced visceromotor responses of the rACC to colorectal distension, whereas lesions of the rACC or microinjections of antagonists of the AMPA or NMDA receptors into this region normalized the visceromotor responses in rats with colorectal inflammation [130]. Manipulations in the rACC did not alter the normal responses to colorectal distension [130]. Rats without inflammation showed conditioned place aversion to colorectal distension which was abolished by microinjection of NMDA or AMPA antagonists into the rACC or by rACC lesions [131]. The visceromotor responses were not affected by these manipulations [131]. Rats trained to expect visceral pain in a passive avoidance paradigm showed increased regional cerebral blood flow in the ACC as well as the prelimbic cortex and amygdala [132]. These results indicate that the rACC is important to the affective responses to pain and to contextual pain memory [131, 132].

In addition to coding the unpleasantness of physical pain, the dorsal ACC is also active in the pain of social rejection or of emotional loss [133]. Imaging studies showed volunteers participating in studies where they are made to feel excluded or viewed negatively showed activity in the dorsal ACC and the anterior insula correlating with the strength of the emotion [133]. Some individuals would rate “social pain” on the same scale as previous instances of physical pain [134]. A consensus is emerging that there are interactions among these regions, such that a distressing emotional state can increase activity in the somatosensory regions to increase pain perception. In one study, participants were subjected to functional MRI scanning while looking at images of an ex-partner or a close friend and while thinking of their emotions associated with the breakup or the friendship [135]. The same individuals were also subject to painful thermal stimuli applied to the volar forearm for 15 sec [135]. There was considerable overlap in regions stimulated by physical pain and emotional pain, including the dorsal ACC and the anterior insula, but also the sensory thalamus and the SII region [135]. Other studies showed SII activation in response to observing others in pain [136]. It is particularly interesting that an individual with CIP, and thus with no inputs to the somatosensory regions, reported feeling pain for the first time after the death of a close relative [137].

Although imaging studies show that numerous brain regions are activated by pain, the identification of a specific “pain cortical area” has been elusive [138]. The groundbreaking work pioneered by Wilder Graves Penfield continued by his colleagues at the Montreal Neurological Institute identified the function of cortical areas by using focal electrical stimulation of conscious patients undergoing brain surgeries, generally in order to identify epileptogenic foci. These studies produced significant mapping of somatosensory and motor functions, but did not identify a pain center, and the idea that there no specific “pain cortex” persisted for years [138]. Focal electrical stimulation of the primary somatosensory cortex (SI) produces somatosensory sensations such as paresthesiae and temperature changes, but not pain [139]. However, recent studies with electrical stimulation of the dorsal posterior part of the insular cortex produced localized pain [140]. An effort to determine the somatotopic organization of this region found that discrete stimulation produced nonsomatosensory responses such as parasthesiae, warmth, vertigo, fear, and anxiety and produced pain when applied to the posterior two-thirds of the insula [139, 141]. The pain was described as burning, stinging, “pins and needles,” headache, or similar to muscle cramping or crushing [141]. A somatotopic organization with varying receptive fields (from 0.5% to 50% of total skin area) was described [141], which is consistent with the somatotopically organized inputs from the posterior part of the ventral medial nucleus (VMpo) of the thalamus [142, 143]. Importantly, the insular cortex projects to its counterpart in the contralateral hemisphere, which helps explain the bilateral stimulation-evoked pain as well as the development of bilateral pain with nerve injury [141]. An extensive cortical mapping study performed over 12 years with 164 patients and 4160 stimulation sites revealed that stimulation-evoked pain was rare (1.4%) and occurred only when stimulation was applied to the posterior and upper part of the insula and the medial part of the SII area [138]. This region may represent a somatotopically organized “pain cortex,” as other regions activated by pain do not produce pain upon stimulation [138]. An analyses of temporal analyses of somatosensory evoked potentials suggests that the insula is a likely “generator” of pain perception [144]. This interpretation is supported by a case study of a patient with an epileptogenic focus in the posterior 1/3 of the right insula [145]. The individual had daily seizures lasting seconds to minutes that were extremely painful [145]. The sensations would begin as unpleasant tingling parasthesiae that would progress to burning and electrical-like sensations and become a throbbing sensation before resolving within a few minutes. Electrical stimulation of the same region produced similar sensations, and thermocoagulation of the insular seizure focus resolved the painful seizures [145]. High frequency discharges and high voltage spikes were recorded from the adjacent secondary somatosensory cortex (SII) and the midcingulate cortex with a short latency, which may possibly contribute to the pain sensations [145]. Other than the insula, the SII is the only other brain region that produced painful sensations when stimulated [139].

The view that the posterior insula and the SII area may integrate pain is supported by several studies employing imaging or stimulation. MRI studies show that the dorsal insular cortex is activated by pain as well as temperature changes and other interoceptive modalities, and that it is active in patients with chronic pain and when allodynia is evoked in patients with neuropathic pain [142, 146]. Studies performed with recording electrodes in the SII and application of laser thermal stimuli to discrete locations on the body showed a somatotopic representation of somatosensory and pain sensations in the SII [147]. The SII neurons showed an ability to encode sensation from threshold to just beyond the onset of a painful sensation, but did not encode for intensities above this ceiling [147], suggesting that this region may provide fine-grain discrimination of stimulus intensity up to painful levels [147]. In contrast, neurons of the insula did not encode sensations below the pain threshold, but encoded intensity of painful stimuli without reaching a saturation point [147]. Patients with lesions of the posterior part of the insular cortex showed elevated pain thresholds to thermal and mechanical stimuli applied to the contralateral hand, whereas subjects with lesions of the anterior insula did not exhibit altered pain thresholds [148]. A later examination of 2 patients with poststroke lesions that included the insula described both individuals as having elevated ratings of pain intensity to noxious stimuli, elevated activity of SI, and they retained the ability to discriminate pain intensity, although there was no evidence of insular activity [149]. However, it should be noted that the individuals had damage that included the anterior and posterior insula and parts of SII [149]. The higher pain ratings might have been due to a loss of the insula's ability to process pain affect and intensity with regard to context [149].

Studies performed in primates showed the insula receives its thalamic input from a “dedicated” nociceptive pathway. Laminae I projections reach the VMpo of the thalamus, which contains topographically organized clusters of neurons responsive only to nociceptive or thermal stimuli, indicating that this nucleus was specific for pain and temperature sensation [143, 150]. The VMpo of the thalamus sends projections to the insula, providing a somatosensory nociceptive input to this region. Whereas the VMpo does not exist as a distinct subnucleus in the rat, the caudal part of the VPL of the rat was found to be an analogous region that likely differentiated into the subnucleus in primates [151]. However, chiefly because of its communication with the amygdala, the insula also participates in the motivational/affective aspects of pain [150]. Imaging studies have shown that the insula encodes the intensity and laterality of pain [152]. Moreover, the “unpleasantness” of tonic pain was highly correlated with insular activity [153]. It is now accepted that the insula is important to both discriminatory and motivational/affective aspects of pain [150], and convergence of the medial and lateral pain system [154]. Interestingly, loss of pain and thermal sensation through the VMpo results in disinhibition of the lamina I pathway that projects via the medial dorsal (MD) thalamic nucleus to the ACC [143]. This is consistent with the dysesthetic burning pain surrounding an area of analgesia and thermanesthesia in patients with thalamic pain syndrome due to lesions of the VMpo [143].

### 3.3. Descending Pathways

The demonstration of descending systems that modulate nociceptive input from the periaqueductal grey (PAG) is probably one of the most important contributions to our understanding of pain, as this spurred considerable efforts in the exploration of pain modulatory pathways. Tsou and Jang (1964) found a profound antinociceptive effect when morphine was microinjected into the PAG of the rabbit [155]. Subsequent studies with electrical stimulation of the PAG also revealed a profound antinociception in rats [156]. The electrical stimulation of the PAG was rapidly adapted to relieve intractable pain in man and is commonly cited as one of the most rapid applications of an experimental finding to clinical application [157–159]. Its reversibility by naloxone indicated the recruitment of endogenous opioidergic mechanisms [157]. Curiously, PAG stimulation has also led to development of severe migraine-like headaches [160]. Deep brain stimulation of the PAG is used in patients with intractable pain, including neuropathic, postamputation, plexopathies, anaesthesia dolorosa, or poststroke pain, but these patients are carefully selected [161, 162]. Descending pain modulatory systems are depicted in Figure 1(b).

Numerous animal studies have since shown that the PAG is a source of descending opioid-mediated inhibition of nociceptive inputs [163–165]. The PAG receives nociceptive inputs from the spinal cord through connections with the parabrachial nucleus [166]. Imaging studies in human volunteers have shown that the PAG responds to pain [167, 168]. “Offset analgesia,” where a painful stimulus followed by a more intense stimulus appears less painful when the intense stimulus is terminated, is associated with increased PAG activity in imaging studies [169]. Electrophysiologic and pharmacologic studies performed with animals and imaging studies in human volunteers also showed that electroacupuncture analgesia involves the activation of the PAG [170]. The PAG receives inputs from cortical regions, including the rACC, and likely mediates “top-down” endogenous pain inhibition arising from more rostral sites [171, 172]. Activation of this region by engaging cortical sites is likely the mechanism through which placebo analgesic effects (discussed below) are mediated [172, 173]. Neuroanatomical studies revealed that the PAG sends projections to noradrenergic pontine nuclei and the rostroventromedial medulla (RVM), resulting in inhibition of nociceptive inputs at the level of the spinal cord by the release of norepinephrine and serotonin [163, 174, 175]. Disinhibition of these projections from the PAG by endogenous or exogenous opioids activate this descending system to produce antinociception from the PAG [176]. However, it is not clear if the PAG projections directly communicate with the noradrenergic and serotonergic descending fibers, or if this communication is polysynaptic, acting through intermediate relays [175, 177].

### 3.4. Bidirectional Pathways

Along with the inputs from the PAG, the RVM also communicates with the noradrenergic nucleus locus coeruleus and the thalamus and is considered to be the final common relay in descending modulation of nociceptive inputs [178]. Numerous early studies showed that electrical stimulation or morphine microinjection in the RVM produced antinociception in animal models [178]. The RVM sends projections to the dorsal horn of the spinal cord and to the trigeminal nucleus caudalis and forms synapses with interneurons or second-order neurons that send ascending nociceptive projections [179–181]. Several electrophysiologic and behavioral studies indicate that the RVM produces “bidirectional” pain modulation, in that it can inhibit or enhance nociceptive inputs [179]. Low levels of RVM stimulation facilitated, and higher levels of stimulation inhibited, nocifensive responses in the rat [182]. This property of the RVM may play a significant role in endogenous pain inhibitory systems as well as maintenance of enhanced abnormal pain states [179]. Microinjection of lidocaine into the RVM inhibited neuronal firing of dorsal horn neurons in response to electrical and natural stimulation in normal and nerve-injured rats, suggesting that the predominant influence from the RVM is facilitatory, and that descending facilitation is enhanced by peripheral nerve injury [183]. Microinjection of lidocaine into the RVM of rats abolished hyperalgesia during naloxone-precipitated withdrawal [184], hyperalgesia after hindpaw incision [185], experimental pancreatitis [186], hindpaw injection of formalin [187], and blocked development of latent sensitization after prolonged opioid exposure [188]. Nonselective blockade of excitatory synapses with kynurenic acid prior to peripheral nerve injury blocked the development of behavioral signs of neuropathic pain in rats [189]. Lidocaine in the RVM also blocked behavioral signs of tactile allodynia in animal models of migraine [190] and medication overuse headache [191]. A review of several animal models of chronic pain found differential activation of descending inhibition and facilitation [192]. Animal models of inflammation demonstrated a preponderance of inhibition over facilitation, corresponding to an attenuation of hyperalgesia, whereas formalin and neuropathic models demonstrated increased facilitation relative to inhibition and enhanced hyperalgesia [192]. In a recent study, it was found that approximately one-half of Holtzman rats would develop behavioral signs of tactile allodynia and hyperalgesia, whereas the remainder would show normal responses to thermal and tactile stimuli after peripheral nerve injury [188]. Blockade of RVM activity with lidocaine blocked signs of neuropathic pain in nerve-injured rats with tactile allodynia and unmasked the same signs of enhanced abnormal pain in the rats with nerve injury but no allodynia, suggesting that the presence of descending inhibition of nociception protects against development of enhanced pain states [188]. These results are consistent with the concept that neuropathic pain and dysfunctional pain states may occur because of a deficit in endogenous descending pain inhibitory systems [116, 171, 193–195].

A especially fascinating finding resulting from electrophysiological studies performed in the RVM of lightly anesthetized rats was the discovery of neurons with activity that correlated with nociceptive responses to noxious radiant heat [171, 196, 197]. Neurons that increased firing immediately prior to the tail-flick response were labeled “on-”cells, and those that paused firing immediately prior to the reflex were labeled “off-”cells, while neurons with no detectable changes in activity were labeled “neutral” cells [171, 196, 197]. Important insights into the nature of descending modulatory circuitry came from studies by Fields and colleagues in which activity of neurons in the RVM were paired with a behavior elicited by a noxious stimulus (i.e., the tail-flick response to noxious heat) in lightly anesthetized rats [198–200]. Both the off-cells and on-cells were found to project to the spinal dorsal horn, indicating that they may exert modulatory influences on nociceptive inputs [197, 201].

Opioids administered systemically or by direct microinjection into the PAG or the RVM results in a disinhibition of the off-cells and consequently cause a marked increased activity of these neurons [178, 202]. Disinhibition of off-cells is considered to be “necessary and sufficient” for antinociception [178, 202]. The off-cells can be inhibited by application of GABA into the RVM and disinhibited by GABA antagonists, indicating that they are likely modulated by inhibitory GABAergic interneurons or by GABAergic projections from the PAG [203–205]. Activation of the PAG with the nonsteroidal anti-nflammatory metazinol (dipyrone) attenuated nociceptive responses of dorsal horn neurons, and this effect was reversed by GABA administered into the RVM, suggesting the antinociceptive relay in the RVM depends on blocking a GABA-mediated inhibition of the descending inhibitory neurons [206]. In addition, the off-cells are directly inhibited by activation of the *κ*-opioid receptor [207]. Microinjection of the *κ*-opioid agonist U69593 into the RVM unmasked behavioral signs of neuropathic pain in rats with peripheral nerve injury but without allodynia by blocking descending inhibition [188].

The on-cells of the RVM are directly inhibited by opioids, and are activated by cholecystokinin (CCK) via the CCK_2_ receptor [171, 208, 209]. Immunohistological studies for protein and mRNA revealed considerable colocalization of CCK_2_ receptors with *μ*-opioid receptors on RVM neurons which show characteristics of pain facilitation cells and may correspond with on-cells [210]. Microinjection of CCK into the RVM produces behavioral signs of enhanced pain and elicited release of PGE_2_ in the spinal CSF, whereas a CCK_2_ antagonist administered into the RVM blocks behavioral signs of allodynia and hyperalgesia in a model of nerve injury [211, 212]. Selective ablation of RVM neurons expressing the CCK_2_ or the *μ*-opiate receptor was accomplished by internalizing administering CCK or the *μ*-opiate agonist dermorphin conjugated to the cytotoxin saporin [210]. Ablation of these facilitatory RVM neurons prevented and reversed the expression of behavioral signs of tactile allodynia and thermal hyperalgesia in rats with peripheral nerve injury, but only after a period of 7 days after the injury [210]. Enhanced behavioral responses were present even with the pretreatment, suggesting that descending facilitation maintains the late phase of chronic pain, but does not mediate the early phase, which is presumably driven by enhanced excitability of nociceptors [210]. Microinjection of lidocaine into the RVM also abolished evidence of ongoing or nonevoked pain in nerve-injured rats [213].

The neurochemical nature of these descending projections is unsettled and remains somewhat controversial. The RVM includes the n raphe magnus and the n gigantocellularis pars alpha, which contain serotonergic neurons that project to the spinal cord [214, 215]. Approximately 20% of RVM neurons that project to the spinal dorsal horn are serotonergic, and the remainder are likely glycinergic or GABAergic [216, 217]. Serotonin is released in the spinal cord in response to stimulation of the PAG or the RVM [218]. Studies with intrathecally injected serotonergic antagonists indicate that activation of some of the 5-HT subtypes (5-HT_2A_ and 5-HT_3_) facilitates nociception whereas activation of the 5-HT_1A_, 5-HT_1B_, 5-HT_1D_ and the 5-HT_7_ subtypes is inhibitory [104, 114, 219–223]. Electrical stimulation of the motor cortex produced antinociception to noxious thermal stimuli in rats with peripheral nerve injury, and this antinociceptive effect was blocked by the GABA agonist muscimol administered into the RVM or by spinal injection of a 5-HT_1A_ antagonist [224]. This experiment suggested that descending inhibition from the RVM was associated with serotonin acting at the 5-HT_1A_ receptor in the spinal cord [224]. The spinal administration of a 5-HT_7_ antagonist blocked the antinociceptive effect of morphine microinjected into the RVM, whereas that of a 5-HT_3_ antagonist blocked hyperalgesia induced by CCK administered into the RVM, leading to conclusions of inhibitory and facilitatory activity, respectively, through these receptors [225]. Consistent with this conclusion, systemic administration of 5-HT_7_ agonists blocked capsaicin-induced hyperalgesia in mice, whereas 5-HT_7_ antagonists elicited mechanical hypersensitivity [226]. The 5-HT_7_ receptor has been identified in the DRG and on central terminals of primary afferent fibers [227, 228] as well as on GABAergic interneurons in the dorsal horn of the spinal cord [227], which is consistent with a role in pain modulation [226]. 

An early electrophysiologic and immunohistochemical study of 25 identified RVM neurons found that none of the on-cells or off-cells were serotonergic and only 4 neutral cells showed label for 5-HT [229]. However, all 3 types of RVM neurons expressed serotonergic apposition [230]. Some investigators suggested that the RVM on-cells and off-cells are modulated by serotonergic neutral cells [231, 232]. Electrophysiologic studies provided evidence that descending inhibitory or facilitatory projections can be either serotonergic or GABAergic [233]. Studies where serotonergic neurons of the RVM were selectively depleted of serotonin by locally injected SiRNA in rats with inflammation indicate that the serotonergic RVM neurons mediate facilitation, and not inhibition, of nociception [234]. However, other studies show that the projections from the PAG synapse with spinopetal GABAergic neurons [180]. Retrograde tracer studies also show that spinally projecting neurons express GABA, glycine, or both [235], and electrophysiologic studies showed that descending GABAergic or glycinergic projections inhibit noxious inputs into the dorsal horn of the spinal cord [216]. Electrophysiologic studies employing juxtacellular recording techniques and filling techniques allowed for the identification and labeling of on-cells, off-cells, and neutral cells in the rat [229]. It was found that the large majority of the off-cells and of the neutral cells expressed GAD67, thus were likely GABAergic [236]. Just over one-half of on-cells were also determined to likely be GABAergic [236]. The remainder of these RVM neurons was determined to be neither GABAergic nor serotonergic, as serotonin was found only in a subset of the neutral cells [236]. The influence of the RVM on spinal dorsal horn and trigeminal neurons may differ, as the neurons that project from the RVM to these regions show differences in synaptic connections and neurochemistry [181]. The spinopetal RVM projections formed synaptic connections with dendrites and soma, and approximately two-thirds expressed GAD67, whereas only one-third of those projecting to the trigeminal dorsal horn expressed GAD67, and these axons formed synapses mostly with dendrites [181].

Emerging evidence suggests that the functionality of the on-, off-, and neutral cells is variable in magnitude and direction, suggesting considerable plasticity of responses. Peripheral inflammation resulted in a conversion of neutral cells into both on-cells and off-cells [237]. Several studies revealed that responses of RVM neurons to noxious heat do not predict the responses to visceral stimuli [238–240]. In one study, neutral cells defined by thermal stimuli exhibited on-cell or off-cell types of responses to colorectal distension (CRD) [238]. A subsequent study that characterized RVM neuronal responses to CRD or heat found that only one-third of the responsive cells responded in the same direction to both stimuli [239]. In addition, some neurons responded by excitation or inhibition to nonnoxious CRD and in the opposite direction to noxious CRD [239]. Similar findings were reported by Dickenson and colleagues [240]. They found that there was no correlation between neuronal activity after somatic stimulation and after CRD [240]. For example, on-cells identified somatically could appear as on-, off-, or neutral cells after visceral stimulation [240]. However, pregabalin, which attenuated the visceromotor responses to CSD, also inhibited the activity of the subset of RVM neurons that responded as on-cells to CRD or heat [240]. These results suggest that the composition of RVM neurons is likely more complex than previously thought, with subclasesses of the 3 classes of RVM neurons [238–240]. Importantly, however, the concept that RVM on-cells are pronociceptive is still supported [240].

In addition to a serotonergic component, early studies showed a strong noradrenergic component to supraspinal antinociception arising from the PAG and RVM. Electrical or chemical stimulation of the PAG or RVM produced antinociception along with release of norepinephrine into the spinal CSF [218, 241]. Antinociception from electrical stimulation or opioids applied in the RVM or the PAG was attenuated by the spinal administration of noradrenergic antagonists [242–246]. Descending noradrenergic mediation of supraspinal opioid-induced antinociception is mediated through activation of spinal *α*_2_ adrenergic receptors acting presynaptically, inhibiting neurotransmitter release, and postsynapticcally, by hyperpolarizing dorsal horn neurons [242, 244, 247]. Spinally administered *α*_2_ adrenergic agonists produce a strong antinociceptive effect and synergize with opioids to produce marked enhancement of antinociception [247–250]. In contrast, activation of excitatory spinal *α*_1_ adrenoceptors enhances neuronal responses to nociceptive inputs, although some of these neurons are inhibitory GABAergic interneurons that may contribute to antinociception [242, 251]. Clinical studies have shown that epidural administration of the *α*_2_ adrenergic agonist clonidine produces effective pain relief in patients with neuropathic cancer pain [252]. Furthermore, spinal, but not systemic, clonidine blocked capsaicin-induced pain in normal, healthy volunteers [253].

The pontine noradrenergic nuclei A5, A6 (locus coeruleus), and A7 (Kölliker-Füse) give rise to the descending noradrenergic projections to the spinal cord [174, 254–256]. Selectively labeling of only the spinopetal noradrenergic projections was performed with adeno-associated viral (AAV) vector encoding green fluorescent protein (GFP) and driven by the PRSx8 promoter, which codes for the dopamine *β*-hydroxylase (DBH) gene [254]. Projections from A6 were found throughout the spinal cord, but with the greatest concentration in the cervical and lumbar dorsal horns, whereas A7 projections were found throughout the lumbar cord and the ventral horn, and A5 projections were chiefly localized to the thoracic intermediolateral cell columns and around the thoracic sympathetic preganglionic neurons [254]. Electrical stimulation of A6 releases norepinephrine in the spinal dorsal horns [257] and attenuates behavioral responses to thermal nociceptive stimuli [258].

The RVM receives noradrenergic inputs from the A6 and A7 regions and sends enkephalinergic and substance p-expressing projections to A7 [256, 259, 260]. The enkephalinergic projections to A7 are capable of producing a bidirectional modulation of spinal nociceptive inputs, with inhibition of nociception mediated by the spinal *α*_2_ adrenoceptors and a pronociceptive effect driven by spinal *α*_1_ adrenoceptors [260]. Both A6 and A7 also receive inputs from the PAG that terminate in apposition to DBH-immunoreactive and nonimmunoreactive dendrites and soma [174]. The A6 noradrenergic nucleus also receives projections from the RVM and the amygdala as well as thalamic nuclei and the insular cortex [261]. The interactions among the cortical sites, the descending noradrenergic system, and the PAG-RVM pathway can produce a bidirectional control of nociceptive inputs [262].

Enhanced activity of descending noradrenergic systems in chronic pain states may represent an effort to modulate the enhanced pain state. Some noradrenergic neurons of A6 express the NK1 receptor, and substance P microinjected into the A6 produces marked reversal of behavioral signs of neuropathic pain in rats with peripheral nerve injury [263]. The antiallodynia mediated by substance P in the A6 is blocked by NK1 antagonists or spinal administration of the *α*_2_ adrenergic antagonist yohimbine [263]. Peripheral nerve injury increases responses of A6 neurons to noxious somatic stimuli [258]. Inhibition of descending noradrenergic projections by use of adenoviral vectors to transfect the noradrenergic neurons resulted in enhanced hyperalgesia and responses to nociceptive inputs as well as an increase in biochemical markers of enhanced neuronal activity in rats with nerve injury or inflammation [264]. Descending noradrenergic projections were likely mediating an inhibitory effect on enhanced nociception in these conditions [264]. Peripheral nerve injury results in upregulation of norepinephrine synthesis in A6 and in a remarkable increase in noradrenergic nerve terminals in the spinal dorsal horn, which accounts for the enhanced antinociceptive activity of noradrenergic agonists in the nerve-injured state [265]. The antinociceptive effect of spinal clonidine was markedly enhanced in mice with diabetic neuropathy, which also provoked a significant upregulation of *α*_2_ adrenergic receptors in the spinal dorsal horn [266]. Taken together, these studies provide strong evidence for the potential utility of therapeutics that can enhance *α*_2_ adrenergic activity in the management of chronic, and especially neuropathic, pain states.

The engagement of descending noradrenergic systems in pain modulation has important clinical implications. Many of the drugs currently showing clinical efficacy in neuropathic or dysfunctional pain states act at least in part through noradrenergic mechanisms. The drugs tramadol and tapentadol are notable examples. Based on its fairly low affinity for the *μ*-opiate receptor, tramadol would normally be expected to show little analgesic efficacy [267, 268]. However, tramadol also blocks the neuronal reuptake of norepinephrine and of serotonin, and its analgesic efficacy is the result of a synergistic interaction between its opioid activity and the activation of spinal *α*_2_ adrenergic receptors subsequent to elevated spinal levels of norepinephrine [267–269]. Tramadol is efficacious and well tolerated for mild-to-moderate neuropathic, postsurgical, and acute pain [268, 270, 271]. Tapentadol, derived from tramadol, shows greater affinity for the *μ*-opiate receptor and also produces its enhanced analgesic through the synergistic interaction between the *μ*-opiate and *α*_2_ adrenergic mechanisms [268, 272, 273]. Accordingly, it also has an opioid sparing effect, and results in greater patient compliance because of less intense gastrointestinal side effects [268, 274, 275]. Tapentadol is efficacious and well tolerated against severe pain and is used against moderate-to-severe postsurgical, neuropathic, and cancer pain [268, 272–275].

Selective and mixed monoamine reuptake inhibitors also show efficacy in inflammatory, neuropathic, and dysfunctional pain states, largely due to enhanced noradrenergic activity [276–278]. The mixed serotonin/norepinephrine reuptake inhibitor duloxetine was the first to be approved by the US FDA for treatment of pain from diabetic neuropathy [276, 279]. Since then, it has also shown analgesic efficacy against pain in fibromyalgia and osteoarthritis [280, 281]. Recently, the mixed serotonin/norepinephrine reuptake inhibitor milnacipran also showed efficacy in some patients with fibromyalgia [282].

The gabapentinoids gabapentin and pregabalin show clinical efficacy against neuropathic and fibromyalgia pain [283, 284], and emerging evidence suggests that a noradrenergic component may also play a role in its efficacy. Microinjection of gabapentin into the A6 of rats with peripheral nerve injury abolished behavioral signs of abnormal pain that was blocked by spinal administration of the *α*_2_-adrenergic antagonist idazoxan [285]. Spinal or systemic gabapentin also blocked incision-induced hyperalgesia in rats and was reversed by spinal idazoxan [286]. Finally, studies in human volunteers showed that gabapentin elevated spinal CSF levels of norepinephrine in surgical patients and reduced the opioid requirement for postoperative pain relief [286].

The finding that activation of the PAG can produce remarkable antinociception in animals, followed by the discovery of RVM neurons with differential responses to nociception, leads to an explosion of research, and is dedicated to understanding the neuroanatomy and physiology of pain and its modulation. Accordingly, the PAG-RVM-spinal axis has been extensively studies over the past 4 decades. More recently, significant advances in imaging technologies led to a wealth of new information obtained from imaging studies performed in humans, especially within the past decade. Consequently, our conception of pain modulation has evolved from a rather linear mechanism descending from the PAG to that of a complex pain matrix that includes the SI, SII, insula, anterior cingulate cortex, prefrontal cortex, thalamus, amygdala, and the PAG and RVM [116, 193]. The cortical sites are activated by ascending nociceptive inputs and are responsible for processing these inputs into the somatosensory and motivational/affective components of the pain experience. However, the interactions among cortical and subcortical sites can also modulate pain in an inhibitory or facilitatory manner [116, 193, 287]. Since these regions are also important to other functions, such as emotion, motivation, and attention, they play a key role in modifying the pain response based on context [116, 193, 287].

An example of how these sites may interact to alter pain perception is demonstrated by the phenomenon of placebo analgesia. Early studies showed that approximately 33% of the population is responsive to placebo-induced analgesia [288]. Placebo analgesia may be explained in part by the considerable overlap among components of the pain matrix and the mesolimbic reward circuitry [289]. The ACC, insula, amygdala, ventral tegmental area (VTA), and the nucleus accumbens (NAc), which are components of the mesolimbic reward system, are also implicated in pain processing [289, 290]. Moreover, the ACC and the amygdala have projections to the PAG and RVM and thus can influence descending modulation of nociception [179, 182, 291, 292]. Neuroimaging techniques have now established that the placebo analgesic response is likely mediated by activation of these midbrain structures [116, 193, 293]. Placebo-induced analgesia is abolished by naloxone, indicating the activation of endogenous opioidergic systems [294]. The use of positron emission tomography (PET) scans with [^11^C]-carfentanil revealed activation of *μ*-opioid receptors in the ACC, anterior insula, and the dorsolateral prefrontal cortex during the placebo response [295]. Using combinations of warmth, noxious heat, placebo, and remifentanil injections, a significant covariance between ACC and PAG activity was found during placebo, but not the pain only, condition [296]. Naloxone abolished placebo analgesia and reduced the activity of the PAG and RVM as well as the placebo-induced coupling between the ACC and the PAG [297]. These results show that placebo-induced analgesia is mediated through the endogenous opioidergic descending pain inhibitory system [297].

Studies with fMRI showed that the expectation of pain relief reduces pain perception as well as the activity of the ACC and insula [298]. Subjects that received painful thermal or electrical stimuli prior to and after application of a cream were trained to expect analgesia by reducing the intensity of the stimulus [298]. In a second session, where the stimulus intensity was not reduced after cream application, the subjects reported a reduction in pain intensity that was accompanied by reduced activation of the ACC and insula [298]. Similar results were seen when patients with irritable bowel syndrome were used [299]. In a study where PET and [^11^C] raclopride and fMR were used to image dopamine levels in individuals that received placebo infusion followed by a 20 period of expectation of a painful stimulus, those individuals that reported greater pain relief showed increased dopamine in the NAc [300]. These results show activation of the mesolimbic reward pathway during anticipation of analgesia [300]. Healthy volunteers conditioned to expect pain relief displayed placebo-induced analgesia [301]. Fear conditioning was performed by introducing the anticipation of electrical shock, which abolished placebo analgesia [301].

The term “nocebo” describes an effect opposite to placebo; that is, the expectation of a worsening outcome in response to a treatment [302]. Volunteers that were preconditioned to expectation of pain relief with ketorolac and subsequently informed that they would receive a hyperalgesic agent reported enhanced pain [303]. Preconditioning stimuli or verbal instructions to condition the individual to expect increased pain resulted in reports of pain to nonpainful stimuli as well as enhanced pain in response to noxious stimuli [304]. In a study designed to isolate expectancy of pain, subjects received visual cues to indicate if a high or low noxious thermal stimulus was to be applied, but they always received the high stimulus [305]. This procedure revealed changes in the ipsilateral ACC, the head of the caudate, cerebellum, and the contralateral cuneiform nucleus (nCF) [305]. It was reasoned that increased pain expectancy activates a pain network that modulates afferent input at the level of the nCF [305].

In addition to modulating placebo and nocebo responses, the overlap between the areas responsive to pain and those that are components of the reward pathway are consistent with observed interactions between emotional state and pain perception [306]. The opponent process theory states that changes from homeostasis result in activation of an opponent processes in order to restore homeostasis, and that the rapid termination of the initial process should result in a sensation, that is, the converse (i.e., opposite valence) of, and less intense than, the initial sensation [290]. Under this theory, the pleasantness, or value of the “reward,” should increase as the efficacy of the opponent process to restore homeostasis increases, thus providing strong motivation to return to the homeostatic state [290]. Accordingly, application of a painful stimulus should be followed by a sensation of relief when the stimulus is terminated, and that the sensation of relief should increase as the noxious stimulus increases [290]. Put simply, pain relief is rewarding. The visual analog scale (VAS) ratings given by healthy volunteers receiving noxious thermal stimuli at increasing intensities that was terminated abruptly showed that the intensity of relief was less than the intensity of the pain felt, but increased as pain increased in intensity [290]. The individuals then rated the intensity and the perceived pleasantness on return to baseline temperature or to innocuous cooling. The cooling condition significantly increased the intensity and the level of pleasantness felt compared to the baseline condition, even though he temperatures applied were the same [290]. This study demonstrated that pain relief is more than simply a reduction in pain intensity, but is an opponent process involving the reward mechanisms [290]. In a study where subjects were trained to anticipate a painful stimulus, those that scored higher for dread and pessimism indicated greater pain intensity and greater relief on pain offset [307]. Imaging performed in that study revealed that the pleasantness of pain relief correlated with activity of the NAc [290]. In addition to mediating pleasure, the NAc also signals unexpected reward and positive or negative prediction error indicated by opposite valences [307]. In an imaging study performed with healthy volunteers and individuals with chronic back pain, VAS scores to pain intensity and to pain relief (pleasantness) were taken along with fMRI scanning [308]. Both groups rated pain intensity similarly, but the CBP patients also noted significant relief of their back pain during the noxious thermal stimulus [308]. Whereas the healthy control group indicated pleasantness with offset of the noxious stimulus, the CBP patients indicated “unpleasantness” (i.e., negative VAS scores) [308]. However, thermal pain offset was rated pleasant when the CBP patients were concentrating on the back pain [308]. These responses correlated with phasic NAc activity recorded with stimulus offset. Increased activity was seen with pain offset in healthy volunteers and in CBP patients when they concentrated on the back pain; otherwise, NAc activity of opposite valence was observed in CBP patients [308]. The phasic NAc activity serves as a reward prediction signal, as the phasic changes precede the pain offset that indicate impending reward in healthy volunteers and impending punishment in CBP patients [308]. It is possible that the NAc signal associated with pain offset could be used as an objective marker of chronic pain [308].

The interaction between pain and reward circuitries led to the “Motivational-Decision Model,” which is described in terms of negative, aversive and positive, or appetitive, motivational systems [289]. In a context when both a reward and pain are present, the 2 processes are prioritized based on homeostatic state which presents greater survival advantage versus reward magnitude [289]. Thus, a strong motivation escape from danger should be antinociceptive, and that anything that is more important (e.g., intense pain) than a given reward should diminish the value of that reward [289]. Numerous studies showed that appetitive stimuli, such as food, music, sex, or erotic images diminish pain [289]. This model was demonstrated in a study where cues indicating a high or low probability of winning a monetary reward were presented along with the prospect of noxious electrical stimulation [309]. The prospect of pain rendered monetary gain less desirable, and this was accompanied by decreased activity of the ACC and ventral striatum, which includes the NAc, which is consistent with the involvement of these regions in the integration of cost and benefit [309]. In addition, stronger pain-related signals from the insula to the orbitofrontal cortex correlated with greater pain avoidance at the cost of little or no reward, which was indicative of individual variability in the influence of pain on choices made relative to potential reward [289, 309]. The complex interplay between reward and pain processing is important in attempts to understand the increased pain sensitivity and attenuated reward processing seen in some chronic pain patients [309].

### 3.5. Diffuse Noxious Inhibitory Controls (DNIC)

Early observations that the electrophysiologic responses of dorsal horn neurons to somatic noxious stimuli were inhibited when a second noxious stimulus was applied to an extrasegmental site led to formulation of the concept of diffuse noxious inhibitory control (DNIC) [310, 311]. Generally, the dorsal horn neurons that were modulated by DNIC were the convergent WDR neurons that receive inputs from peripheral A*β* and C-fibers. Moreover, DNIC was abolished by systemic naloxone or spinal cord section, indicating the involvement of descending modulation from supraspinal sites [310–312]. Electrophysiologic studies indicate that DNIC is integrated at the level of the dorsal reticular nucleus (DRt), which receives ascending nociceptive inputs, communicates with the PAG, RVM, thalamus, amygdala, and cortical sites, and also sends pain modulatory projections to the spinal cord [313–317]. Since a single DRt neuron can send axonal projections to several sites in the CNS, this region can engage pain modulation through several mechanisms and can form a spinal-supraspinal-spinal feedback loop that modulates pain [318, 319].

Converging evidence suggests that dysfunctional chronic pain syndromes may be caused, at least in part, by deficiencies in DNIC [320]. DNIC can be expressed experimentally in normal healthy individuals by applying a heat probe delivering constantly increasing temperature to the hand (experimental pain stimulus) and then placing the foot in noxious-cold water (conditioning pain stimulus) and observing a decrease in the perceived intensity of the esperimental pain stimulus (i.e., the heat ramp) [194]. Performing the same protocol in patients with irritable bowel syndrome (IBS) or temporomandibular disorder (TMD) produced increased, rather than decreased, sensitivity to the noxious heat ramp, indicating an absence of DNIC [194]. Enhanced pain sensitivity in place of DNIC was also demonstrated with patients with fibromyalgia [321]. Additional studies found deficient DNIC in patients with osteoarthritis of the knee [322], chronic pancreatitis [323], rheumatoid arthritis [324], and long-term trapezius myalgia [325]. A recent electrophysiological cortical mapping study was performed to detect temporal changes in the activity of different brain regions that may contribute to DNIC [326]. Application of a conditioning stimulus (hot water bath) lowered reported pain scores to the experimental stimulus (noxious heat pulses) and increased activity of the amygdala and orbitofrontal cortex, followed by reductions in activity of the SI, SII, ACC, and insula [326]. Activation of the amygdala and orbitofrontal cortex during the conditioning stimulus may have inhibited the activity of the latter regions to produce DNIC [326]. Imaging studies also showed reduced activity of the insula, SI, and ACC when DNIC was evoked in normal, healthy volunteers, but increased pain and increased, instead of decreased, activity of these regions during the conditioning stimulus in patients with IBS [327]. The inclusion of continuous cognitive visual tasks during the conditioning stimulus in this study indicated that the reduced reported pain scores in the normal individuals were not a result of distraction caused by the conditioning stimulus [328]. In addition to describing a mechanism through which dysfunctional pain states may emerge, the examination of DNIC may also have practical clinical uses as well. The risk of enhanced postsurgical pain in patients was predicted by attenuated expression of DNIC [329, 330].

The development of chronic tension-type (CTT) headache and migraine headache may also be due to a reduced capacity for DNIC. Patients with CTT receiving conditioning stimuli (noxious thermal heat to the thigh) did not report reduced pain scores to the experimental noxious stimulus (electrocutaneous noxious stimulus to the arm or temple), whereas DNIC was evident in normal healthy volunteers [195]. Similar results were obtained by employing an occlusion cuff as the conditioning stimulus [331]. Patients with migraine with aura showed significantly reduced activity of the orbitofrontal cortex and increased activity of pain matrix regions when compared to control volunteers [332]. Decreased orbitofrontal cortex activity may result in disinhibition of the other regions and promote migraine [332]. Additional studies revealed that patients with or without aura have a deficit of DNIC [333]. These studies strongly indicate that dysfunctional pain states, generally arising with no obvious injury or etiology, may be due to a dysfunction of endogenous pain modulatory systems.

## 4. Summary

The discovery that analgesia could be initiated from electrical stimulation of the brain was signal moment in pain research. This discovery precipitated a robust effort that over the years produced a wealth of information to help us understand how pain is initiated, perceived, and modulated. Since that time, we have come to understand that specialized nociceptors, when activated, send inputs into the CNS through the spinal or medullary dorsal horns that are then transmitted to sites in the medulla, midbrain, and cortex. These ascending projections activate descending pain modulatory mechanisms that project through the midbrain PAG and the RVM to either facilitate or inhibit further nociceptive inputs. Because of significant advances in neuroimaging techniques that allow functional analyses with a greater degree of spatial and temporal resolution, our perceptions of how pain is integrated and modulated in the central nervous system have evolved tremendously especially over the past 2 decades. From the early linear system of pain modulation from the PAG through the RVM and descending to the spinal cord, the concept of a complex “pain matrix” that includes somatosensory and limbic cortical regions, subcortical elements of the limbic system, the PAG and RVM, and noradrenergic nuclei. The concept of a “top-down” pain modulation system arising from cortical sites has been invoked to explain seemingly disparate phenomena such placebo analgesia, stress-induced analgesia, or pain augmentation as well as the effects of distraction, attention, and emotional state on pain perception. Increasing our understanding of these systems also provides greater insight into the potential mechanisms of action of analgesic drugs and atypical analgesics that are effective in neuropathic and dysfunctional pain states. Finally, these studies lend insight into the mechanisms that may drive dysfunctional pain states, such as the chronic headaches, fibromyalgia, IBS, and others that appear without any discernible injury or pathology. Converging evidence points to altered functioning of endogenous pain modulation, including loss of DNIC, as a potential mechanism driving dysfunctional pain. A greater understanding of the anatomy, physiology, and pharmacology of pain modulatory systems will lead to more advanced therapies against chronic pain states.
